# Partaker: deep-learning-based single-cell-resolution analysis of multi-dimensional, long-term, microfluidic-based time-lapse microscopy data

**DOI:** 10.1093/bioinformatics/btag675

**Published:** 2026-09-10

**Authors:** Henrique H Libutti-Núñez, Bukola A Akindipe, Hamed Rastaghi, Nona Hashemi, Samuel M D Oliveira

**Affiliations:** Department of Nanoengineering, North Carolina Agricultural and Technical State University, Greensboro, NC 27401, United States; Department of Nanoengineering, North Carolina Agricultural and Technical State University, Greensboro, NC 27401, United States; Department of Nanoengineering, North Carolina Agricultural and Technical State University, Greensboro, NC 27401, United States; Department of Nanoengineering, North Carolina Agricultural and Technical State University, Greensboro, NC 27401, United States; Department of Nanoengineering, North Carolina Agricultural and Technical State University, Greensboro, NC 27401, United States

## Abstract

**Motivation:**

Deep-learning segmentation models for microbial time-lapse fluorescence microscopy already exist, but they are often difficult to use consistently across experiments and are not packaged with unified workflows for combining models, quantifying multi-channel fluorescence, and scaling analyses to large microfluidic imaging datasets.

**Results:**

To address these challenges, we developed Partaker, an easy-to-use Python-based graphical tool for deep-learning segmentation, multi-channel fluorescence quantification, and morphological analysis of microbial cells over time. Partaker supports extensible model integration, efficient handling of large imaging datasets, and interactive visualization, enabling time-resolved analysis of population fluorescence distributions from per-cell measurements. We demonstrate performance using a two-strain validation experiment (housekeeping and inducible) and show robust recovery of population-level fluorescence dynamics with single-cell resolution.

**Availability and implementation:**

Partaker is implemented in Python and is available on GitHub (https://github.com/SamOliveiraLab/partaker). A versioned release of the software is archived on Zenodo (https://doi.org/10.5281/zenodo.18844425). Documentation, example datasets, and installation instructions are provided in the repository.

## 1 Introduction

Microscopy has become an essential tool for understanding cellular dynamics (Stephens and Allan 2003). The advent of fluorescent reporters has enabled researchers to engineer cells that express tagged proteins, revealing gene expression and protein localization in real time (Tsien 1998).

In time-lapse experiments, researchers must integrate fluorescence data with morphological information to comprehensively describe bacterial cell behavior (Young *et al*. 2011, Martins *et al*. 2018). Microfluidics has emerged as a key technology in this context, allowing the fabrication of microscale environments that mimic natural conditions. These systems support applications ranging from “organ-on-a-chip” and microbial interaction studies to synthetic ecology and high-throughput bioengineering.

Nevertheless, microfluidic microscopy experiments remain data-intensive. They generate large, multidimensional image datasets that are prone to drift, uneven illumination, and noise. Image files can easily reach hundreds of gigabytes, complicating downstream analysis.

**Figure 1:**
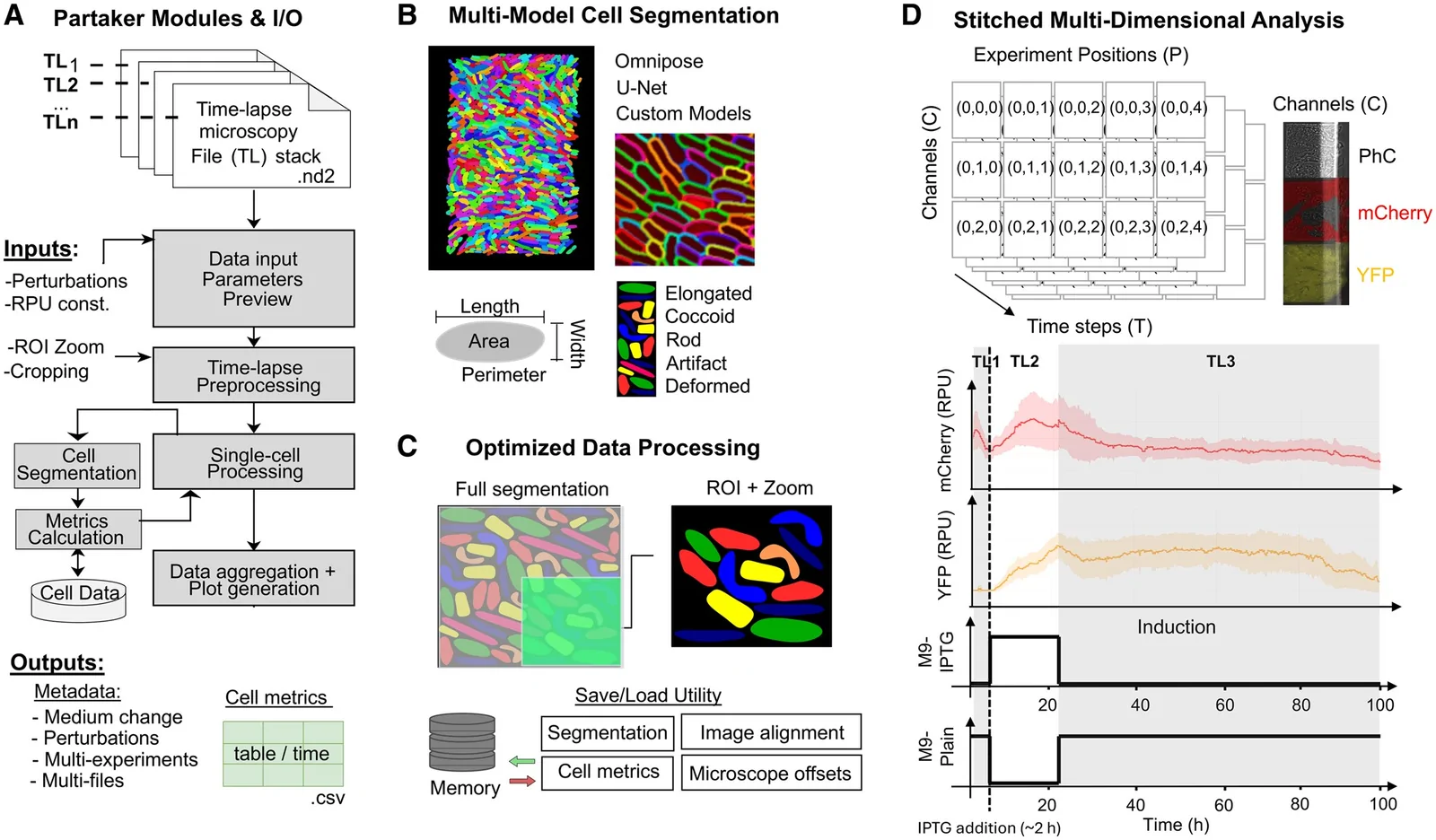
Overview of multi-channel analysis via Partaker. (A) In Partaker, multiple ND2 files containing time-lapse microscopy data are used as data and the user inputs experimental parameters such as medium perturbations, RPU calibration values, and visual regions-of-interest. The software seamlessly concatenates these files on the time axis. (B) Multiple segmentation models (Cellpose, Omnipose, and U-Net variants) can be selected to optimize cellular detection across different imaging situations. The cell segmentation result is used to extract cell metrics (C) Computational optimizations include polygon-based ROI, and state preservation allowing interrupted analyses to be saved and resumed. (D) Single-cell fluorescence measurements are aggregated into temporal profiles synchronized with medium perturbations represented as step functions. Stitched Multi-Dimensional Analysis refers to temporal concatenation and synchronized analysis of sequential ND2 acquisition files across channels, positions, and timepoints. Data from sequential acquisition files is automatically integrated along a continuous time axis.

To address these challenges, we developed Partaker, a Python-based software package for cell segmentation, single-cell fluorescence quantification, and morphological analysis (Fig. 1). By combining these features, Partaker enables systematic studies of microbial community dynamics in microfluidic environments. Detailed descriptions of the graphical interface, data processing, and analysis are provided in Supplementary Methods S1 and Supplementary Figs S1–S3, available as supplementary data at *Bioinformatics* online.

Several tools have since advanced automated bacterial image analysis. DeLTA 2.0 (O’Connor *et al*. 2022) employs a two-step U-Net pipeline for mother machine segmentation and tracking. SuperSegger (Stylianidou *et al*. 2016) and its successor OmniSegger (Lo *et al*. 2025) provide segmentation-to-lineage pipelines for rod-shaped bacteria. BacStalk (Hartmann *et al*. 2020) focuses on stalked and budding bacteria using classical morphological methods. FAST (Meacock and Durham 2023) combines Omnipose segmentation with downstream fluorescence and growth analysis. mAIcrobe (Brito *et al*. 2025) uses lightweight convolutional networks for real-time segmentation. MiSiC (Panigrahi *et al*. 2021) trains a single U-Net to generalize across species and imaging modalities using synthetic phase-contrast data. MicrobeJ (Ducret *et al*. 2016), widely used for morphometric analysis, has recently integrated Omnipose-based deep learning segmentation alongside its classical image-processing algorithms. Partaker was specifically developed to address challenges commonly encountered in long-term microfluidic time-lapse microscopy experiments. These challenges include microscope drift, illumination artifacts, multi-position image acquisition, and the need to simultaneously quantify multiple microbial populations across fluorescence channels. To address these issues, the platform integrates image registration for drift correction, ROI-based filtering of user-defined capture regions, morphology extraction, and temporal aggregation within a single analysis workflow. Partaker also supports multi-channel fluorescence quantification in calibrated Relative Promoter Units (RPU), accepts both ND2 and TIFF image formats, and provides access to multiple segmentation backends, including Omnipose, Cellpose 3, DeepBacs, and a custom U-Net model. Together, these capabilities facilitate consistent downstream analysis of large microscopy datasets without requiring users to reconstruct workflows across multiple software packages or perform extensive manual data processing.

## 2 Implementation

### 2.1 Deep learning cell segmentation

Partaker implements deep learning-based segmentation that can leverage multiple model architectures. Users can choose from a list of pretrained models or integrate their own, facilitating reproducibility and domain adaptation. The modular framework separates the segmentation logic from the data handling pipeline, simplifying model substitution. Common architectures include U-Net, originally developed for biomedical image segmentation, and Cellpose, a generalist algorithm capable of segmenting cells from diverse image types without extensive retraining. An overview of segmentation pipeline is shown in Fig. S2, available as supplementary data at *Bioinformatics* online.

### 2.2 Multi-channel fluorescence analysis

The analysis module processes multi-channel microscopy data to distinguish bacterial populations based on fluorescence signals. In the present experimental design, fluorescence channels were used to distinguish bacterial populations and quantify reporter activity. For each segmented cell, Partaker extracts fluorescence measurements including mean fluorescence intensity, integrated fluorescence, and calibrated Relative Promoter Unit (RPU) values. These measurements are computed on a per-cell basis and can be aggregated across populations to generate time-resolved fluorescence profiles. This step ensures each cell’s fluorescence profile is associated with its corresponding population identity and experimental role. Single-cell and population-level fluorescence analysis are shown in Fig. S4, available as supplementary data at *Bioinformatics* online.

### 2.3 Morphological analysis

The following set of morphological descriptors is extracted from segmented masks: area, perimeter, equivalent diameter, orientation, aspect ratio, circularity, solidity, and extent. These measurements, when combined with fluorescence data, provide rich datasets for characterizing cell health and growth dynamics. The resulting dataset enables advanced downstream analyses, such as phenotype clustering or growth-rate modelling.

Partaker exports these morphological measurements alongside fluorescence data on a per-cell, per-frame basis, enabling users to perform correlation analyses, track morphological shifts across experimental conditions, and integrate results with external statistical or machine learning pipelines. For instance, changes in cell aspect ratio may coincide with gene expression bursts, and spatial patterns of fluorescence can reveal communication networks within bacterial populations. By tracking these morphological features over time, Partaker facilitates the investigation of how physical cell properties interact with genetic regulatory circuits.

### 2.4 Software architecture and performance

Partaker combines direct data access for rapid operations with a publish/subscribe system for long-running or asynchronous tasks. This hybrid approach optimizes responsiveness and scalability. There are three main services responsible for implementing the program’s core functionality: *MetricsService*, which merges morphological and fluorescence data using the pandas library for efficient computation; *SegmentationService*, which bridges segmentation requests and models; and the *GUI*, which provides an interactive interface for visualization and analysis. Expanded views of individual processing stages and user interface elements are provided in Supplementary Figs S1–S3, available as supplementary data at *Bioinformatics* online.

### 2.5 Test experiment

Partaker was validated using a two-strain co-culture composed of an IPTG-inducible YFP reporter strain and a constitutive mCherry housekeeping-reference strain grown within the same microfluidic-based environment and confined to monolayer chambers (Fig. 1D). Cells were cultured under a constant flow of M9 medium, and IPTG was introduced at the indicated induction time point to activate YFP expression. Phase-contrast and multi-channel fluorescence time-series images were acquired at multiple *x*–*y* locations corresponding to individual monolayer chambers over the course of the experiment (Fig. 1). Fluorescence measurements were converted to Relative Promoter Units (RPU) using a standard calibration plasmid assayed under identical experimental conditions (see Supplementary Information, available as supplementary data at *Bioinformatics* online).

Images were acquired every 5 minutes for approximately 100 hours (Figs 1D and S7, available as supplementary data at *Bioinformatics* online). Imaging was performed on a fully automated inverted microscope (Nikon Eclipse Ti2-E PFS) equipped with a 100× oil-immersion APO objective and a Perfect Focus System for long-term focal stability. Multi-channel fluorescence excitation was provided by an LED-based epifluorescence illumination system (X-Cite Xylis) using manufacturer-specified filter sets: a Semrock GFP filter set (GFP-4050B) for YFP and a Semrock TRITC filter set (LED-TRITC-A) for mCherry. Images were recorded with a Dhyana 400D 4.2 MP monochrome sCMOS camera at 2048 × 2044 pixels per frame.

## 3 Results

Segmentation performance was evaluated on 45 manually corrected instance masks sampled from the two-strain validation experiment (Section 2.5), spanning four chamber positions (P0, P4, P5, P6) at 11–12 time points per position distributed from approximately 0.4 h to 92 h of the 100-hour experiment (1200 phase-contrast frames acquired at 5-minute intervals). Ground-truth masks were initialized with omnipose_bact_phase and manually corrected by a trained annotator, and the custom U-Net was trained on a separate experiment (80 phase-contrast frames, four positions) with no overlap with this evaluation set. Using instance-level matching at IoU ≥0.5 on raw images, bact_phase_cp3 (Cellpose 3) achieved the highest independent accuracy (JI = 0.610 ± 0.215, mean matched IoU = 0.858 ± 0.035, Dice = 0.734 ± 0.178); omnipose_bact_phase scored higher (JI = 0.978 ± 0.022, IoU = 1.000 ± 0.000, Dice = 0.989 ± 0.012) but is an upper bound, as it generated the initial ground-truth masks. These values are lower than those commonly cited for these backbones (e.g. DeepBacs U-Net IoU >0.85 and Omnipose IoU >0.9), largely as a metric-definition effect: we report an instance-level Jaccard Index at an IoU ≥0.5 matching threshold, which additionally penalizes missed and spurious detections in dense monolayers and is therefore not directly comparable to the pixel-level IoU quoted in the literature. The corresponding mean matched IoU (0.858 for bact_phase_cp3; Table S1, available as supplementary data at *Bioinformatics* online) is consistent with those published pixel-level values. A modern competitor, mAIcrobe (Brito *et al*. 2025), reached a comparable JI = 0.610 ± 0.271 (Dice = 0.718 ± 0.243) using Cellpose 4 model, confirming that segmentation accuracy is driven by the underlying model rather than the platform. Full dataset composition and the evaluation protocol are provided in Table S1, available as supplementary data at *Bioinformatics* online and Section 1.4.1.

The Intersection over Union (IoU) metric is defined as:


(1) $$\text{IoU}=\frac{\text{Area}\;\text{of}\;\text{Overlap}}{\text{Area}\;\text{of}\;\text{Union}}$$

The Dice coefficient is defined as:


(2) $$\text{Dice}=\frac{2\times \text{Area}\;\text{of}\;\text{Overlap}}{\text{Area}\;\text{of}\;\text{Predicted}+\text{Area}\;\text{of}\;\text{Ground}\;\text{Truth}}$$

In both equations, the overlap represents correctly segmented pixels, the union corresponds to the total pixels in either mask, and the predicted and ground-truth areas denote the total pixels in their respective masks.

The Jaccard Index (JI) is defined as:


(3) $$\text{JI}=\frac{TP}{TP+FP+FN}$$

where *TP*, *FP*, and *FN* denote the numbers of true-positive, false-positive, and false-negative cell detections, respectively. A predicted cell was considered a true positive when its Intersection over Union (IoU) with a ground-truth cell was at least 0.5. Predicted cells that could not be matched to a ground-truth cell were counted as false positives, whereas ground-truth cells without a matching prediction were counted as false negatives.

To compare single-cell and bulk fluorescence analysis, we evaluated RPU measurements derived from segmentation-based per-cell analysis against pixel-averaged bulk calculations (Fig. S7, available as supplementary data at *Bioinformatics* online). Partaker successfully segmented cells across *1200* frames over the 100-hour experiment. Single-cell analysis computed RPU values for each segmented cell, with the mean and standard deviation reflecting biological variability across the population. In contrast, bulk analysis calculated RPU from mean field-of-view intensity, where variability represents pixel-level noise rather than cell-to-cell heterogeneity. Over the 100-hour experiment, segmentation-based quantification reduced background contributions and yielded higher, more representative promoter activity values. Histogram analysis suggested population-level shifts in fluorescence distributions over time rather than uniform intensity increases across all cells. These histograms qualitatively illustrate heterogeneous fluorescence responses across the population during the experiment. These results demonstrate that single-cell analysis preserves biological heterogeneity and captures spatiotemporal transitions that are obscured by bulk pixel-averaged measurements.

## 4 Conclusion

We present Partaker, an open-source, Python-based tool for time-resolved fluorescence and morphological analysis of microbial communities. Its modular design supports extensibility for diverse segmentation and analysis workflows in synthetic biology and microbiology research. By addressing issues of dataset scale, fluorescence variability, and analysis reproducibility, Partaker bridges the gap between microscopy data acquisition and quantitative community-level interpretation.

## Supplementary Material

btag675_Supplementary_Data

## Data Availability

The custom U-Net was trained on the phase-contrast co-culture-2 dataset (80 frames from four positions) and evaluated on the independent co-culture-1 dataset (45 manually corrected frames), with no overlap between the training and evaluation sets. Both datasets are available in the Partaker Segmentation Benchmark Zenodo repository (https://doi.org/10.5281/zenodo.20577330). The pretrained cellpose_deepbacs backend integrated in Partaker derives from the DeepBacs *Escherichia coli* bright-field dataset (https://doi.org/10.5281/zenodo.5550935). The experimental microscopy dataset consists of multi-channel time-lapse ND2 files acquired from a two-population bacterial community grown in microfluidic chambers; in this study we analyzed the first 100 hours (1,200 frames). It is publicly available in the BioImage Archive under accession S-BIAD3015 (https://doi.org/10.6019/S-BIAD3015).
