# Supplementary material for: Partaker: deep-learning-based single-cell-resolution analysis of multi-dimensional, long-term, microfluidic-based time-lapse microscopy data
Source: Bioinformatics. 2026 Sep 10;42(9):btag675. doi: 10.1093/bioinformatics/btag675 (PMC13592469; doi:10.1093/bioinformatics/btag675)
Supplement: Supplementary file 1 [file btag675_supplementary_data.docx]

**Supplementary Information for “Partaker: deep-learning-based single-cell-resolution analysis of multi-dimensional, long-term, microfluidic-based time-lapse microscopy data.”**

Henrique H. Libutti- Nunez^1^, Bukola Akindipe^1^, Hamed Rastaghi^1^, Nona Hashemi^1^, and Samuel M.D. Oliveira^1,*^

^1^Department of Nanoengineering, North Carolina Agricultural and Technical State University, Greensboro, NC, USA 27401

*Corresponding author: smdoliveira@ncat.edu

Table of Contents

[1. Supplementary Methods	2](#_Toc231820099)

[1.1 Graphical User Interface	2](#_Toc231820100)

[1.2 Software Components	3](#_Toc231820101)

[1.3. Installation and Requirements	5](#_Toc231820102)

[1.4 Data Processing Pipeline	5](#_Toc231820103)

[1.4.1 Segmentation Model Performance Comparison	6](#_Toc231820104)

[1.4.2 Comparative Analysis with Existing Tools	8](#_Toc231820105)

[1.4.3 Machine Learning Evaluation Protocol	9](#_Toc231820106)

[1.5 Data Analysis Outputs	9](#_Toc231820107)

[1.6 Use-Case: Long Experiment Population Dynamics Analysis	10](#_Toc231820108)

[1.7 Implementation Details	10](#_Toc231820109)

[1.7.1 Image Registration Algorithm	10](#_Toc231820110)

[1.7.2 Morphological Feature Extraction	11](#_Toc231820111)

[1.7.3 Fluorescence Quantification Techniques	13](#_Toc231820112)

[1.7.4 Memory Management for Large Datasets	13](#_Toc231820113)

[1.7.5 Memory Benchmark	13](#_Toc231820114)

[1.8 Cell Analysis vs Bulk Analysis	14](#_Toc231820115)

[1.9 Bacterial strain, growth conditions, and induction	15](#_Toc231820116)

[1.10 Microscopy	16](#_Toc231820117)

[1.11 Microfluidic Device	16](#_Toc231820118)

# **1. Supplementary Methods**

## **1.1 Graphical User Interface**

Partaker's Graphical User Interface (GUI) is a single window with three tabs: Segmentation, Population, and Morphology. Each tab displays the working data and controls (**Figure S1**).

The Segmentation tab serves as the primary interface for dataset exploration and cell segmentation configuration. It incorporates multi-dimensional navigation controls that enable sequential traversal through temporal coordinates (T), spatial positions (P), and fluorescence channels (C). This interface supports comprehensive dataset inspection before computational analysis. The display mode selector toggles between original microscopy data and segmented representations, allowing immediate visual assessment of segmentation quality. For batch processing, the tab includes parametric controls for defining the temporal interval and position selection, which determine the scope of automated segmentation. This modular design enables selective processing of specific experimental subsets, optimizing computational resource allocation.

The Population tab implements fluorescence dynamics analysis functionality through temporal parameter selection and channel-specific visualization. The interface allows precise definition of the analysis time range and selection of fluorescence channels for quantitative assessment. The analytical engine aggregates single-cell fluorescence measurements across the defined parameters to generate population-level fluorescence profiles. These profiles reveal temporal dynamics of reporter protein expression within microbial communities. The visualization component employs statistical plots that display both mean trends and population heterogeneity through appropriate variance indicators. This analytical approach enables quantitative assessment of intercellular communication dynamics in synthetic bacterial consortia.

The Morphology tab provides morphometric analysis capabilities, including temporal profiling and dimensionality reduction visualization. The interface visualizes cellular morphological parameters over time, revealing physical adaptations to environmental conditions or genetic modifications. Additionally, the tab implements principal component analysis (PCA) visualization for individual time points, reducing the multidimensional morphological feature space to 2 dimensions for intuitive visual interpretation. This representation highlights morphological heterogeneity within bacterial populations and enables the identification of distinct phenotypic subpopulations. The combination of temporal morphometric profiling and frame-specific PCA visualization provides a comprehensive analytical framework for investigating bacterial morphological dynamics in microfluidic environments.

The integrated design of these three analytical modules enables a sequential workflow from image segmentation to population-level fluorescence dynamics to morphological characterization, facilitating comprehensive single-cell-resolution analysis of bacterial communities in microfluidic environments.

**Figure S1 Graphical User Interface-** The GUI in Partaker is organized in a single window and three main tabs. The first view (A) depicts the full application window, with the image preview on the left-hand side, as well as controls for time, location and channel, while the right part contains the “Segmentation” section which contains controls for segmenting intervals of the dataset. The (B) “Morphology” section allows the user to visualize a PCA plot and the table for cell morphology metrics at a given frame. The (C) “Population” tab displays single cell fluorescence plots, allowing for the visualization reporters along the time axis, paired with medium step functions.

## **1.2 Software Components**

The application is structured into three primary components: data management, data analysis, and the user interface. These components interact through a publisher-subscriber (pub/sub) messaging pattern, which decouples operations and enables asynchronous processing of computationally intensive tasks (**Figure S2**).

The core data management is implemented through the ImageData singleton class, which provides centralized access to time-lapse microscopy data across the application. The class implements methods to retrieve raw and processed images, handle segmentation requests, and apply image transformations such as registration and region-of-interest (ROI) cropping.

Analytical services in Partaker are implemented as modular components with specific responsibilities. The SegmentationService integrates deep learning models with a caching mechanism to optimize performance for repeated analysis of the same frames, which commonly occur when users visually compare segmentation results with the original phase contrast frame. In our current release, we have developed a 5-level U-Net trained on the co-culture-2 dataset and integrated Cellpose.

**Figure S2 Segmentation pipeline-** The code for Partaker has been structured into several modules with specific responsibilities each. The “Front-End” modules are responsible for the GUI, while the “Back-end” section implements the services that perform bacterial segmentation, data exporting and compute the single cell fluorescence and morphological features.

To optimize performance, Partaker implements a custom caching mechanism through the SegmentationCache class, which stores segmentation results in memory-mapped arrays. This approach minimizes memory usage while providing fast access to previously processed frames. The cache is structured by model type, allowing users to switch between different segmentation approaches without recomputing results.

The GUI is built with PySide6 (Qt for Python) and organized into specialized widgets for different analytical functions. The application implements a tab-based interface with dedicated panels for segmentation, population analysis, and morphological measurements. Each UI component subscribes to relevant events from the data and analytical services, updating its display in response to changes in the underlying data.

Persistence uses a combination of JSON serialization for metadata and HDF5 storage (via h5py) for large datasets, such as segmentation masks. This approach balances human-readable configuration files with efficient binary storage for volumetric data. The application supports saving and loading complete analytical states, allowing users to pause and resume their work across sessions without recomputing resource-intensive results.

## **1.3. Installation and Requirements**

Partaker requires Python 3.11 (>= 3.11, < 3.12) and runs on Apple Silicon macOS (arm64), Windows (x86_64), or Linux (x86_64/aarch64, glibc >= 2.28). Intel macOS and older Linux (glibc < 2.28) are not supported and will not install. The software can be installed from the GitHub repository (<https://github.com/SamOliveiraLab/partaker>) using the following commands:

bash

git clone https://github.com/SamOliveiraLab/partaker.git

cd partaker

uv venv

source .venv/bin/activate # On Windows: .venv\Scripts\activate

uv pip install -e.

Partaker uses environment variables for configuring a test dataset and the trained weights for the U-Net segmentation model. The environment variables are PARTAKER_ND2_TEST and PARTAKER_UNET_WEIGHTS, and can, for example, be configured in an env.sh file:

export PARTAKER_ND2_TEST="~/partaker/1-SR_1_9_6hPre-C_1h30aTc_TS_MC2.nd2"

export PARTAKER_UNET_WEIGHTS="~/partaker/checkpoints/partaker_unet.pt"

After installation, you can launch the application using uv run partaker_app. While Partaker can run on CPU-only systems, GPU acceleration is recommended for efficiently processing large datasets. The software has been tested with CUDA-compatible NVIDIA GPUs and Apple Silicon GPUs.

For development purposes, configuration templates for VS Code and PyCharm are provided in the documentation. These templates include the necessary environment variables and debug configurations for development and testing.

## **1.4 Data Processing Pipeline**

The Partaker workflow implements a sequential image processing pipeline optimized for bacterial cell analysis in microfluidic environments. The software operates on the ND2 file format, which is widely used in many microscopy imaging systems because it supports multidimensional data storage. The nd2 library with Dask integration enables efficient handling of these complex data structures.

Upon experiment initialization, the user interface presents the microscopy data with three primary navigation parameters: temporal (T) for progression through the time series, positional (P) for selection between spatial locations within the microfluidic device, and channel (C) for transitioning between different imaging modalities such as phase-contrast and fluorescence channels.

Before cell segmentation, the software offers an image registration module to correct mechanical drift that commonly occurs during long-term time-lapse experiments. The registration algorithm identifies stable structural features within the microfluidic chambers and computes positional offsets between consecutive frames. These displacement vectors are subsequently applied to all frames, ensuring spatial consistency throughout the time series. The registration process uses Numba-accelerated functions to optimize performance.

The Region of Interest (ROI) selection module provides a polygon-based interface for users to delineate specific analysis regions (**Figure S3**). The interface allows sequential point placement to form a polygon boundary with functionality for polygon reset, completion, and acceptance. OpenCV's computational geometry algorithms convert the defined polygon into a binary mask, creating a spatial filter for subsequent segmentation. This mask excludes cells outside the ROI or intersecting its boundary, which is critical for eliminating partial-cell artifacts.

**Figure S3 ROI-** A demonstration of the ROI selection process (A versus B). Starting from (A) a suboptimal segmentation, (C) the user selects a polygon in the “ROI Dialog” window, and subsequently (B) cells outside of the region are discarded in all subsequent segmentations. The discarded cells are not considered for fluorescence analysis or morphological feature extraction.

The boundary-aware filtering mechanism enhances single-cell measurement accuracy by combining morphological and set-theoretic operations. Specifically, binary dilation from *scipy.ndimage* generates a boundary zone, which is then used to identify and exclude cells that contact it, thereby preventing morphological bias from partially visible cells.

Cell segmentation is performed using the selected deep learning model, with visualization options including: a normal display of raw microscopy data; a segmented view showing grayscale masks; an overlay presentation with contour highlighting; and a labeled segmentation with diverging colorization for individual cell identification. The overlay visualization incorporates channel-specific color transformations that distinguish between phase contrast and different fluorescent protein markers (mCherry, YFP), with parameterizable contour thickness for optimal visual assessment.

**1.4.1 Segmentation Model Performance Comparison**

Partaker integrates multiple deep learning architectures to accommodate diverse experimental conditions and image quality variations commonly encountered in bacterial microscopy. The platform currently supports six segmentation models (**Figure S4**) organized across three families: Omnipose (omnipose_bact_phase and omnipose_bact_fluo), which uses distance-field-based mask reconstruction optimized for morphologically diverse bacteria [1], Cellpose 3 (bact_phase_cp3, bact_fluor_cp3, and cellpose_deepbacs), which uses gradient-flow-based mask reconstruction with image restoration capabilities [2]; and a custom 5-level U-Net (unet_custom) trained on the co-culture-2 dataset.

**Figure S4 Segmentation models comparison**- Visual comparison of segmentation outputs across the six integrated models on a representative raw phase-contrast frame. Each panel shows the raw microscopy image alongside model-specific segmentation overlays, illustrating differences in boundary delineation and cell detection across model families. Quantitative accuracy metrics for each model, computed on raw images, are reported in Table S1.

To evaluate the practical performance of the integrated segmentation models, we benchmarked them on 45 manually corrected ground-truth frames from four microfluidic chamber positions (P0, P4, P5, P6), with 11-12 frames per position sampled across the early, intermediate, and late growth phases of the phase-contrast channel. We initialized ground-truth masks with omnipose_bact_phase and manually corrected them with a trained annotator. For each model, we matched predicted instance masks to ground truth using greedy matching at an IoU ≥ 0.5 threshold, and we report the instance-level Jaccard Index (JI), mean matched IoU, and Dice coefficient (**Table S1**).

**Table S1. Performance evaluation**. Instance-level segmentation performance of Partaker-integrated models evaluated on 45 manually annotated phase-contrast frames from four microfluidic chamber positions (P0, P4, P5, P6) spanning the early, intermediate, and late growth phases of a 100-hour E. coli co-culture time course. We computed metrics using greedy instance matching with an IoU threshold >= 0.5. Values are reported as mean +/standard deviation across all evaluation frames (raw images only). Ground truth masks were initialized using omnipose_bact_phase predictions and manually corrected in NAPARI by a trained annotator. The custom U-Net was trained on an independent dataset (P co-culture-2, 80 frames from 4 positions) with no frame, position, or dataset overlap with the evaluation set. *omnipose_bact_phase metrics (JI, IoU, and Dice) represent an upper bound due to circular evaluation: this model generated the initial masks that were subsequently corrected into ground truth. Time/frame is the per-model inference time measured on the same 45 evaluation frames, reported as mean +/- standard deviation, on an Apple Mac Studio (M2 Ultra, 64 GB unified memory); no discrete GPU, inference executed on the Apple Silicon integrated GPU. Model loading and image I/O are excluded, and one warm-up frame per model was discarded.

| Model | JI | IoU | Dice | Time/frame (s) |
| --- | --- | --- | --- | --- |
| omnipose_bact_phase | 0.978+/-0.022* | 1.000+/-0.000* | 0.989+/-0.012* | 0.81 +/- 0.20 |
| bact_phase_cp3 | 0.610+/-0.215 | 0.858+/-0.035 | 0.734+/-0.178 | 0.43 +/- 0.13 |
| cellpose_deepbacs | 0.064+/-0.091 | 0.566+/-0.288 | 0.108+/-0.145 | 0.19 +/- 0.02 |
| Unet_custom | 0.018+/-0.043 | 0.513+/-0.326 | 0.032+/-0.070 | 0.13 +/- 0.00 |
| omnipose_bact_fluo | 0.006+/-0.005 | 0.476+/-0.241 | 0.011+/-0.010 | 0.88 +/- 0.17 |
| bact_fluor_cp3 | 0.005+/-0.005 | 0.441+/-0.239 | 0.010+/-0.011 | 0.32 +/- 0.05 |
| mAIcrobe (Cellpose 4) | 0.610+/-0.271 | 0.849+/-0.062 | 0.718+/-0.243 | n/a |

These results establish several findings relevant to Partaker's multi-model design. Among phase-contrast models, bact_phase_cp3 (Cellpose 3) achieved the highest independent accuracy (JI = 0.610 ± 0.215); omnipose_bact_phase scored higher (JI = 0.978 ± 0.022) but represents an upper bound, as it generated the initial ground-truth masks. The custom U-Net achieved moderate pixel-level detection (IoU = 0.513) but near-zero instance-level accuracy (JI = 0.018), reflecting the limitation of binary segmentation in resolving individual cells in dense monolayers. Fluorescence-trained models (omnipose_bact_fluo, bact_fluor_cp3) scored near zero on phase-contrast data (JI ≤ 0.006), confirming that model selection should match the imaging modality. All values in Table S1 are reported on raw images. This variability is precisely why Partaker provides multiple model options. The instance-level Jaccard Index reported here is not directly comparable to the pixel-level IoU values commonly quoted for these backbones in the literature (for example, DeepBacs U-Net IoU > 0.85 and Omnipose > 0.9): the Jaccard Index additionally penalizes missed and spurious detections under instance matching at an IoU threshold of 0.5, which is particularly stringent in dense monolayers. The mean matched IoU reported in Table S1 (0.858 for bact_phase_cp3) is consistent with those published pixel-level values.

**1.4.2 Comparative Analysis with Existing Tools**

To contextualize Partaker's capabilities within the existing ecosystem of bacterial image analysis software, we compared key features with established tools commonly used in the field: MicrobeJ, Oufti, SuperSegger, and BacStalk. **Table S2** summarizes this comparison:

**Table S2 Comparison of features across bacterial image analysis tools.** Symbols indicate full support (checkmark), partial support (~), or no support (X). Partaker TIFF support is included in the current release. OmniSegger is MATLAB-based but invokes Omnipose (Python) for segmentation. MM = mother machine; 2D = two-dimensional monolayer growth.

| **Feature** | **Partaker** | | **DeLTA 2.0** | | **OmniSegger** | | **BACMMAN** | | **napari-MM3** | | **MiSiC** | | **mAIcrobe** | | **MicrobeJ** | | **Oufti** | | **SuperSegger** | | **BacStalk** | |  |
| --- | --- | --- | --- | --- | --- | --- | --- | --- | --- | --- | --- | --- | --- | --- | --- | --- | --- | --- | --- | --- | --- | --- | --- |
| **Multiple DL seg.** | | **✓** | | **✗** | | **✗** | | ~ | | ~ | | **✗** | | **✓** | | ~ | | **✗** | | **✗** | | **✗** | |
| **Cell tracking** | | **✗** | | **✓** | | **✓** | | **✓** | | **✓** | | **✗** | | **✗** | | **✓** | | **✗** | | **✓** | | **✗** | |
| **GPU accel.** | | **✓** | | **✓** | | ~ | | ~ | | ~ | | ~ | | ✓ | | **✗** | | **✗** | | ~ | | **✗** | |
| **Pop. analysis** | | **✓** | | **✓** | | **✓** | | **✓** | | **✓** | | **✗** | | **✗** | | **✗** | | **✗** | | **✗** | | **✗** | |
| **Model training** | | ✗ | | **✓** | | **✗** | | ~ | | **✓** | | **✗** | | **✓** | | **✗** | | ~ | | **✓** | | **✗** | |
| **Drift correct.** | | **✓** | | ~ | | **✓** | | **✓** | | **✓** | | **✗** | | **✗** | | **✗** | | **✗** | | **✓** | | **✗** | |
| **ROI selection** | | **✓** | | **✗** | | **✗** | | **✗** | | **✗** | | **✗** | | **✗** | | **✓** | | **✓** | | **✗** | | **✓** | |
| **Morphology** | | **✓** | | **✗** | | **✗** | | **✗** | | **✗** | | **✗** | | **✓** | | ~ | | ~ | | **✗** | | **✓** | |
| **ND2 concat.** | | **✓** | | **✗** | | **✗** | | **✗** | | **✗** | | **✗** | | **✗** | | **✗** | | **✗** | | **✗** | | **✗** | |
| **TIFF support** | | **✓** | | **✓** | | **✓** | | **✓** | | **✓** | | **✓** | | **✓** | | **✓** | | **✓** | | **✓** | | **✓** | |
| **RPU calib.** | | **✓** | | **✗** | | **✗** | | **✗** | | **✗** | | **✗** | | **✗** | | **✗** | | **✗** | | **✗** | | **✗** | |
| **Save/resume** | | **✓** | | **✗** | | **✗** | | **✓** | | ~ | | **✗** | | **✗** | | ~ | | **✗** | | **✓** | | **✗** | |
| **Geometry** | | Mono | | MM+2D | | General | | MM | | MM | | General | | Static | | General | | General | | MM | | General | |
| **Platform** | | Python | | Python | | MATLAB | | Java | | Python | | Python | | Python | | ImageJ | | MATLAB | | MATLAB | | MATLAB | |

While several established tools provide robust analysis capabilities for specific use cases, Partaker uniquely combines deep learning-based segmentation, multi-model flexibility, and scalable data management in a unified platform. This integration addresses the growing need for automated, high-throughput analysis of increasingly large microscopy datasets generated by modern long-term time-lapse experiments.

### **1.4.3 Machine Learning Evaluation Protocol**

The custom U-Net (5-level encoder-decoder, 31M parameters, input 512 x 512 x 1) was trained using TensorFlow/Keras with Adam optimizer, boundary-weighted binary cross-entropy loss, and augmentation (rotation, flipping, elastic deformation). Training data: 80 frames from 4 positions of co-culture-2 (Batch 2)—85/15 train/validation split. Early stopping at epoch 70 of 200 (patience 30), achieving 95-96% pixel-level accuracy. The trained Keras weights were converted to a PyTorch state-dict (.pt) for inference; the released Partaker package performs U-Net inference in PyTorch and does not require TensorFlow at runtime. For clarity, the datasets referred to here are: co-culture-2 (Batch 2), an internal phase-contrast experiment from this study used for U-Net training; co-culture-1, a separate internal phase-contrast experiment used exclusively for evaluation; and DeepBacs, an external, publicly available bright-field dataset (Zenodo 5550935) that underlies the pretrained cellpose_deepbacs backend. The custom U-Net was trained exclusively on co-culture-2 from randomly initialized weights; no pretrained weights were loaded, and the DeepBacs dataset was not used for its training or fine-tuning. Training masks were generated with omnipose_bact_phase and used directly as training labels, so the U-Net was trained to reproduce Omnipose-derived labels rather than manual annotations.

The evaluation set (45 frames, 4 positions, co-culture-1) has no overlap with the U-Net training set. All six models were evaluated identically. Instance masks were compared against manually corrected ground truth using greedy IoU matching at threshold 0.5.

## **1.5 Data Analysis Outputs**

Partaker extracts quantitative single-cell data by computationally analyzing segmented microscopy images. For each identified cell, the software calculates morphological descriptors including area (size), major axis length, angular orientation, and solidity coefficient. These parameters, computed using OpenCV and scikit-image functions, serve as quantitative indicators of bacterial growth dynamics and morphological responses to environmental conditions.

Fluorescence quantification is achieved by spatially integrating pixel intensities within each cell-specific mask across corresponding fluorescence channels. This methodology enables precise measurement of reporter protein expression at single-cell resolution. Integrating morphological and fluorescence parameters generates a multidimensional dataset that captures both physical and functional cellular characteristics simultaneously.

The analytical output is structured in a relational database, with entries for temporal coordinates, spatial positions, unique cell identifiers, morphological metrics, and channel-specific fluorescence intensities. This structured data organization is managed using pandas DataFrames and facilitates export to comma-separated value (CSV) format for integration with external statistical software. For larger datasets, memory-mapped Polars dataframes provide enhanced performance. The dataset architecture preserves the hierarchical relationship between population-level trends and single-cell measurements, enabling analysis of intercellular variability and temporal dynamics.

For longitudinal studies, Partaker implements state preservation through HDF5-based serialization of analysis parameters, segmentation results, and region definitions. The segmentation cache utilizes data compression protocols to minimize storage requirements while maintaining data integrity. This architecture enables investigators to suspend and resume analysis sessions without redundant computation, which is particularly advantageous for processing extensive time-lapse datasets.

## **1.6 Use-Case: Long Experiment Population Dynamics Analysis**

A representative application of Partaker is analyzing bacterial communication networks in housekeeping-inducible systems. In this experimental paradigm, multi-channel time-lapse microscopy captures the dynamics of distinct bacterial populations through spatially resolved imaging. Following image registration to compensate for mechanical drift, region selection isolates well-defined microbial communities for subsequent analysis.

Post-segmentation, the software correlates morphological parameters with fluorescence signatures to characterize population-specific phenotypes. Distinct fluorescence channels differentiate between bacterial strains, while temporal analysis reveals intercellular communication dynamics. The resulting quantitative dataset enables precise measurement of signal propagation kinetics, response latency, and phenotypic heterogeneity within microbial consortia.

This analytical methodology is particularly significant for synthetic biology investigations involving genetically engineered bacterial populations that communicate via molecular signaling pathways. By resolving single-cell behaviors within community contexts, Partaker enables the quantitative assessment of emergent population-level phenomena arising from individual cellular responses.

## **1.7 Implementation Details**

### **1.7.1 Image Registration Algorithm**

The image registration algorithm in Partaker implements a tree-search optimization approach to correct for microscope stage drift between consecutive frames in time-lapse sequences. The algorithm identifies stable structural features within the microfluidic chambers and computes optimal frame-to-frame displacements.

The registration process begins with preprocessing through intensity rescaling to normalize brightness variations across the time series. Edge detection identifies the top and bottom boundaries of microfluidic chambers, providing vertical alignment reference points. A row-wise brightness-averaging approach computes the intensity gradient to detect horizontal edges with high precision.

For horizontal and fine vertical alignment, the algorithm uses an optimized Sum of Absolute Differences (SAD) metric to measure the similarity between the reference frame and subsequent images. The alignment search is implemented as a breadth-first tree search that explores potential displacement vectors in eight directions (cardinal and diagonal) and evaluates each candidate shift using the SAD metric. This approach efficiently navigates the displacement space to identify the optimal alignment that minimizes frame-to-frame differences.

Performance optimization is achieved through Numba just-in-time (JIT) compilation, which accelerates the computationally intensive operations, including pixel-wise shifting, SAD calculation, and gradient computation. The algorithm incorporates parallel processing for large frames while maintaining numerical stability through carefully designed reduction operations.

The registration output is a set of frame-specific displacement vectors applied to all subsequent image processing operations, ensuring spatial consistency throughout analysis.

### **1.7.2 Morphological Feature Extraction**

Morphological feature extraction in Partaker employs computer vision techniques to quantify cellular characteristics from segmented masks. The binary masks are first processed to identify individual cell regions using connected-component analysis. For each region, the software applies contour detection to extract the cell boundary. From these contours, the software computes:

- Area: The number of pixels within the cell contour
- Perimeter: The arc length of the cell boundary
- Equivalent diameter: The diameter of a circle with the same area as the cell
- Orientation: The angle between the major axis of the cell and the horizontal axis, determined by fitting an ellipse to the cell contour
- Aspect ratio: The ratio of the major to minor axis lengths, quantifying cell elongation
- Circularity: Calculated as 4π×(area/perimeter²), a measure of how closely the cell resembles a perfect circle
- Solidity: The ratio of cell area to its convex hull area, indicating boundary regularity
- Extent: The ratio of contour area to bounding rectangle area

These metrics serve as quantitative descriptors that the Partaker platform uses to classify segmented cells into five shape-based categories: Artifact, Coccoid, Rod, Elongated, and Deformed. These labels describe geometric appearance rather than physiological state, providing a standardized morphological vocabulary for characterizing bacterial cell shapes across the population.

Classification follows a hierarchical decision tree (**Figure S5**) that evaluates geometric thresholds in sequence. All pixel-based thresholds correspond to an imaging pixel size of 0.0645 µm/pixel (pixel area 0.00416 µm²); approximate physical-area equivalents are given in parentheses below. Artifact refers to objects too small to represent valid cells, typically resulting from segmentation errors or debris (area < 245.5 pixels² (≈ 1.02 µm²) or perimeter < 65.9 pixels). Coccoid cells are small with compact, rounded proportions, consistent with cells that have recently undergone division (area <685.8 pixels² (≈ 2.85 µm²), perimeter <269.2 pixels, aspect ratio < 3.53). Rod describes the typical bacillary morphology expected for E. coli, with balanced circularity, aspect ratio, and solidity values (0.516 ≤ circularity ≤ 0.727, 1.46 ≤ aspect ratio ≤ 3.29, solidity ≥ 0.880). Elongated cells appear as stretched filaments with high length-to-width ratios (area ≥ 2399.0 pixels² (≈ 9.98 µm²), aspect ratio ≥ 5.28, circularity ≤ 0.245). Deformed captures non-standard shapes with low circularity and solidity, indicating deviation from expected bacterial geometry (circularity ≤ 0.589, solidity ≤ 0.706). Therefore, this group of metrics provides different angles for analyzing single-cell masks and offers enough flexibility to ignore parts of the features when the cells are not rod-shaped.

**Figure S5** **Hierarchical decision tree for morphological classification-** Cells are sequentially evaluated against threshold criteria, with the first matching category assigned. Threshold values were empirically determined from E. coli datasets and can be adjusted for other organisms through the platform interface.

Cells that do not meet any specific criteria default to Rod. These thresholds were empirically determined from *E. coli* populations and can be adjusted within the platform for different organisms or experimental conditions. The platform provides real-time visualization of classification results (**Figure S6**). The interface displays individual cells with color-coded labels and allows inspection by cell ID. Population-level dynamics are shown as time-series plots that display both the fraction and the absolute count of each morphology class over the experiment duration, enabling users to track shifts in population composition.

**Figure S6 Morphology classification interface-** Color-coded cell visualizations show Artifact (gray), Coccoid (cyan), Rod (green), Elongated (brown), and Deformed (purple) categories, alongside individual cell inspection displaying metrics and classification for selected cells. Population dynamics plots show the temporal evolution of morphology class fractions and absolute counts across the experimental time course.

### **1.7.3 Fluorescence Quantification Techniques**

Fluorescence quantification in Partaker uses Relative Promoter Units (RPU) calibration to address varying background intensity across experiments. This approach uses reference strains with known fluorescence characteristics to establish a standardized intensity scale.

The RPU calibration process involves measuring the fluorescence of a reference strain under conditions identical to those of the experimental samples. By calculating the ratio of sample fluorescence to reference fluorescence, the system normalizes measurements across different experiments, microscopes, and imaging settings.

To account for spatial variation in background within individual frames, the ROI selection tool allows users to exclude regions with abnormal background characteristics. The cell-specific quantification approach, which integrates intensity values only within the segmented mask area, further mitigates the impact of local background variations on measurement accuracy.

Moreover, fluorescence can be analyzed at multiple ND2 locations for the same time point, allowing the computation of the mean and standard deviation across multiple cells and experimental locations simultaneously.

### **1.7.4 Memory Management for Large Datasets**

Partaker implements a multi-layered memory management strategy to handle large microscopy datasets with numerous positions, time points, and channels. The primary data access layer uses Dask and Xarray via the ND2 library, which provides lazy-loading capabilities for large microscopy files. This approach enables the analysis of datasets that exceed system RAM.

For segmentation results, which can reach significant sizes for long time-lapse sequences, the application implements a custom caching mechanism through memory-mapped arrays. The SegmentationCache class stores segmentation masks in model-specific arrays that persist to disk while maintaining fast random-access capabilities. This approach balances performance with memory efficiency by keeping only the necessary data in RAM.

The metrics data, which combines morphological features and fluorescence quantification, is managed using memory-mapped Polars dataframes. This implementation provides SQL-like query capabilities with minimal memory overhead, allowing for efficient filtering, aggregation, and analysis of large result sets.

The caching strategy maintains a set of indices to track which frames have been processed for each segmentation model, avoiding redundant computation while ensuring consistent results. When saving analysis states, the application uses HDF5 compression to minimize storage requirements while preserving rapid access to previously computed results.

### **1.7.5 Memory Benchmark**

To showcase the software's application, a benchmark was performed using a 7-file, 20-position, 419-frame dataset, totaling 8380 3-channel frames and 68GB on disk. A single ND2 XY position was chosen for processing across the entire time-lapse. The benchmarking machine was a Windows 11 PC with an Intel i5-13500 processor, 64GB of DDR4 RAM, and an Nvidia RTX 3500 GPU. **Table S3** summarizes memory consumption across different execution states.

**Table S3. Memory consumed by Partaker during the time-lapse segmentation process.** Number of frames segmented, the RAM usage, and the number of cells processed.

| Frames Segmented | RAM in use | Cells processed |
| --- | --- | --- |
| 1 | 2659MB | 229 |
| 30 | 1990MB | 6273 |
| 100 | 2550MB | 28445 |
| 170 | 2920MB | 93880 |
| 419 | 4278MB | 339266 |

## **1.8 Cell Analysis vs Bulk Analysis**

This experiment ran for 100 hours, enabling long-term observation of fluorescence dynamics under controlled induction. By applying segmentation-based quantification, fluorescence measurements were restricted to identified cellular regions, reducing contributions from background pixels and non-cellular signal. This reduction in background-driven variability improves the accuracy of calculated RPU values and results in higher, more representative measurements of promoter activity (**Figure S7**).

Because RPU is computed independently for each cell, the analysis preserves biological heterogeneity across the population. Rather than collapsing the signal into a single averaged value per time point, this approach enables tracking of expression states at the level of individual cells. As a result, changes in promoter activity can be evaluated not only in terms of mean intensity but also in terms of distribution structure and population transitions.

The histogram analysis illustrates that fluorescence distributions shift over time across the population rather than increasing uniformly across all cells. These distributions qualitatively demonstrate heterogeneous population-level responses during the experiment and highlight temporal redistribution of fluorescence states across the bacterial population.

Importantly, these transitions can be examined as a function of both time and spatial position, allowing investigation of spatiotemporal effects such as delayed activation, heterogeneous response to induction, or progressive state changes within subpopulations. Such analysis would not be possible with bulk-pixel-averaged measurements, which obscure threshold-based transitions and compress heterogeneous states into a single mean value.

**Figure S7- Comparison of single-cell and bulk fluorescence analysis-** Single-cell analysis (A) quantifies fluorescence at the level of individual segmented cells and converts per-cell intensities to Relative Promoter Units (RPU), with the solid line representing the mean RPU across cells at each time point and the shaded region indicating the standard deviation between cells. Bulk analysis (B) calculates RPU from the mean pixel intensity of the entire field of view, where the shaded region reflects standard deviation between pixels rather than biological units. Histograms of single-cell RPU distributions at representative early, mid, and late time points (C) illustrate population heterogeneity and distribution shifts in single-cell measurements. The induction scheme (D) shows the temporal modulation of IPTG at time-point 2h to drive the observed expression dynamics.

## **1.9 Bacterial strain, growth conditions, and induction**

To validate long-term, multi-channel single-cell quantification under two independent behaviors within the same chamber, two *E. coli* populations were used: (i) an IPTG-inducible YFP reporter population and (ii) a housekeeping reference population with constitutive mCherry expression.

The IPTG-inducible reporter population was *E. coli* HL 1951 (MG1655 background; Addgene bacterial strain #69773), which carries pLacO-1::T710::YFP integrated at the *galK* locus (Addgene #69773) [3].  Cells from this population were induced by introducing IPTG at the specified experimental time point; following IPTG addition, YFP fluorescence is expected to increase (as shown in the representative plots), consistent with inducer-driven reporter activation.

The housekeeping reference population carried pDD103 (Addgene plasmid #119887; RRID: Addgene_119887), which provides constitutive mCherry expression from a pAL5000/pMB1 backbone and kanamycin resistance (Addgene #119887) [4]. This population was not induced; any slow drift in mCherry intensity was treated as baseline variation (e.g., growth state or division-rate effects) rather than an inducible response.

Both populations were loaded and imaged simultaneously within the same chamber, challenging the analysis pipeline to quantify two independent cellular behaviors in independent fluorescence channels: an IPTG-responsive YFP trajectory in the inducible population and a non-induced constitutive mCherry baseline in the housekeeping population. This paired design provides an internal control for long-term tracking and channel-specific quantification.

Overnight cultures were grown in minimal medium (M9) for ~15 h at 37 °C with shaking (250 rpm). Subcultures were prepared by diluting overnight cultures 1:100 into fresh M9 and growing to mid-log phase (OD600 ~ 0.3). For pDD103-containing cells, kanamycin was used as required (50 µg/mL, per plasmid information).  At mid-log phase (or at the experiment-defined time point), IPTG was added to the inducible population, and cells were incubated for the required period before imaging. The housekeeping population was processed in parallel without induction.

## **1.10 Microscopy**

For imaging, cells were loaded into a microfluidic device bonded to a glass coverslip and confined to monolayer chambers. Phase-contrast images were acquired for cell segmentation and time-resolved morphological analysis. Phase-contrast images were captured using a monochrome sCMOS camera (Dhyana 400D, 4.2 MP; 2048 × 2044 pixels per frame).

Fluorescence imaging was performed on a fully automated inverted microscope (Nikon Eclipse Ti2-E PFS) equipped with a 100× oil-immersion APO objective and a Perfect Focus System (PFS) for long-term focal stability. Multi-channel fluorescence excitation was provided by an LED-based epifluorescence illumination system (X-Cite Xylis) using manufacturer-specified filter sets: a Semrock GFP filter set (GFP-4050B) for YFP and a Semrock TRITC filter set (LED-TRITC-A) for mCherry. Phase-contrast and multi-channel fluorescence time-series images were acquired at multiple x–y locations corresponding to individual monolayer chambers over the course of the experiment.

For long-term time-lapse imaging, phase-contrast images were acquired every 5 minutes and fluorescence images every 15 minutes for approximately 100 hours (Figures 1D and S4). Image acquisition was controlled using NIS-Elements (Nikon, Tokyo, Japan).

## **1.11 Microfluidic Device**

Microfluidic experiments were performed using PDMS-based devices containing monolayer chambers connected to a microfluidic channel network. Each device contained 140 monolayer chambers with variable lengths of 40, 45, 50, and 55 µm, a fixed width of 60 µm, and a height of 0.9 µm. This shallow chamber height confined bacterial cells to a single focal plane, allowing long-term single-cell-resolution imaging and fluorescence quantification. The device also contained six parallel main channels supporting two independent flow paths, enabling parallel experiments on the same chip.

The devices were fabricated using standard two-layer photolithography. The first SU-8 layer defined the shallow monolayer chambers, while the second layer defined the main fluidic channels, inlets, and outlets. PDMS was cast on the SU-8 mold, cured, punched to generate inlet and outlet ports, and oxygen-plasma bonded to glass coverslips. Media was supplied through the channel network using syringe pumps under continuous-flow conditions. After cell loading and chamber filling, flow was maintained at 2.5 µL/min during long-term imaging at 37 °C.

Cells were loaded into the device through the inlet channels and confined within the monolayer chambers for time-lapse imaging. The experiments used an IPTG-inducible YFP reporter strain and a constitutive mCherry reference strain to support fluorescence quantification, normalization, and image-analysis validation. Phase-contrast and fluorescence images were acquired over multiple chamber positions and time points using an inverted Nikon microscope. These temporally resolved image datasets were used to validate Partaker’s segmentation, fluorescence quantification, and morphology analysis capabilities.

**References**

1. Cutler, K.J., et al. (2022). Omnipose: a high-precision morphology-independent solution for bacterial cell segmentation. Nature Methods, 19(11), 1438–1448.
2. Stringer, C. & Pachitariu, M. (2025). Cellpose3: one-click image restoration for improved cellular segmentation. Nature Methods.
3. Shin, J.-E., Lin, C. & Lim, H.N. (2016). Horizontal transfer of DNA methylation patterns into bacterial chromosomes. *Nucleic Acids Research*, 44(9), 4460–4471. https://doi.org/10.1093/nar/gkw230.
4. DeLorenzo, D.M., Henson, W.R. & Moon, T.S. (2017). Development of Chemical and Metabolite Sensors for *Rhodococcus opacus* PD630. *ACS Synthetic Biology*, 6(10), 1973–1978. https://doi.org/10.1021/acssynbio.7b00192
